# Research on cause analysis and management of coal mine safety risk based on social network and bow-tie model

**DOI:** 10.1038/s41598-025-15638-w

**Published:** 2025-08-14

**Authors:** Guorui Su

**Affiliations:** Information Research Institute of the Ministry of Emergency Management, 100029 Chaoyang, Beijing, China

**Keywords:** Social network, Bow tie model, Apriori algorithm, Coal mine safety, Cause analysis, Risk control, Energy science and technology, Engineering

## Abstract

Accurate identification of coal mine safety risks is a crucial foundation for mitigating coal mine disasters. This study integrates social network analysis (SNA), the bow-tie model, and association rule mining to systematically analyze safety accident data from a coal mine. A total of 85 causative factors were extracted from 72 accidents and assessed through frequency, marginal influence, and centrality indicators to identify key risk contributors. The bow-tie model was employed to structure these causes into a safety risk control framework based on preventive and mitigation measures. Furthermore, the Apriori algorithm was applied to uncover hidden associations among gas safety risk factors, revealing critical compound relationships among factors such as inadequate safety management, insufficient inspections, high incidence of “three violations”, and poor safety education. The findings indicate that management and human-related factors, particularly the absence of effective safety management systems, safety violations, and inadequate training, are the primary contributors to accidents in coal mines. Consequently, it is imperative to address these issues collectively to ensure effective risk prevention in such environments. The coal mine safety risk causality control model established in conjunction with the butterfly diagram model holds significant theoretical and practical value for coal mine safety production.

## Introduction

Recently, China has made significant progress in coal mine safety, with the death rate per million tons of coal decreasing from 4.94 in 2021 to 0.094 in 2022. This achievement can be attributed to an in-depth analysis of the causes of coal mine safety risks and scientific management. However, the increasing complexity of the coal mining production environment and the chain effects of safety risks are becoming increasingly prominent. At the same time, the high uncertainty of accident causes and conditions has further exacerbated the difficulty of managing safety risks in coal mines^1^. Therefore, identifying the causal factors of accidents in the coal mining process and exploring their underlying causal relationships have become key research topics in China’s coal mine safety studies.

With the gradual deepening of the research on the causes of coal mine safety risks, the relevant theories and practices have also made remarkable progress, providing valuable experience for preventing coal mine accidents and safety management. Zhang^2^ summarized various characteristics leading to gas explosion accidents using statistical analysis, developed a causal mechanism model, and proposed preventive and control measures at the macro and micro levels. Based on accident causation theory, Zhang^3^ conducted an in-depth investigation into the causes of coal mine accidents, providing valuable insights for coal mine safety regulation with regard to the characteristics of coal mine accidents in China. Qiao et al.^4^ adopted the STAMP model to analyze the 12.3 Chifeng coal mine explosion accident and identified flaws in processes such as gas monitoring, ventilation, management supervision, and inter-departmental feedback coordination. Li et al.^5^ analyzed 125 gas explosion accident reports from 2010 to 2020 and used the Carma algorithm to extract 30 major causal factors, finding that violations of operating procedures and ventilation system disruptions were the main causes of the accidents. Liu et al.^6^ conducted a quantitative analysis of coal mine roof accident causation factors based on the Bayesian network, which provides data support for research on accident severity. Zhang^7^ developed a simulation model of the coupling of coal mine accident risk factors based on system dynamics theory, providing a basis for decision-making in coal mine safety risk management. Tan et al.^8^ proposed a new theory for improving coal mine safety management based on qualitative analysis using grounded theory. In addition, researchers have begun to focus on the multidimensional interactions of coal mine accident causation. Wang et al.^9^ investigated the causal mechanisms of coal mine gas explosion accidents by constructing a causal accident network and a dynamic Bayesian model, and found that identifying critical causal nodes and applying targeted network intervention strategies can significantly enhance the effectiveness of accident prevention. Fa et al.^10^ applied text mining techniques and apriori algorithms to analyze 883 coal mine accident reports in China from 2011 to 2020, revealing that mechanical equipment factors, physical environmental conditions, and unsafe preconditions have a direct impact on unsafe behaviors among miners. Fu et al.^11^ developed a novel accident causation model, the 2–4 model, from the perspective of organizational behavior, which precisely deconstructs the accident causes into two primary levels, organizational and individual behavior, and verified its effectiveness in preventing coal mine accidents through the case of Yunnan Private Village Coal Mine. Wang et al.^12^ analyzed 86 reported gas explosion accidents and found that 91% of the unsafe behaviors exhibited by miners were classified as violations. Zhang et al.^13^ studied the interaction relationships of coal mine accident causation using Structural Equation Modeling and the Decision-making Trial and Evaluation Laboratory algorithm. Wang^14^ applied machine learning methods to process coal mine big data and proposed a data-driven accident analysis approach, providing strong support for risk assessment. He et al.^15^ used the Structural Equation Model to conduct a simulation analysis of the coupling relationships of coal mine safety risk causes, scientifically explaining the safety risk pathways and providing a theoretical basis for reducing accident rates.

Above all, the existing studies have achieved significant progress in the theoretical analysis of coal mine accident causation. While some research has utilized methods such as social network analysis to explore the correlation of risk factors, there remains a lack of comprehensive quantitative studies on the interactions among different risk factors and insufficient progress in constructing integrated risk network models. To address these issues, this paper builds a coal mine safety risk causation network based on the theory of SNA and driven by coal mine safety risk information, aiming to effectively measure the causes and perform a quantitative analysis of the interactions between them. Finally, the safety risk management and control bow-tie model based on the leading cause of coal mine safety risks is established to achieve scientific management and control. The research findings have significant theoretical value and practical implications for the safe production of coal mine enterprises.

## Methodology

### Social network analysis

Social network analysis (SNA)^16^ is a quantitative method that uses mathematical and graph theory to model relationships and interactions between social actors. It aims to identify mutual influences within a network and quantify the degree of influence between various factors. A social network can be mathematically expressed as:

1 $$G = \left( {\left( {U,V} \right),E} \right)$$

where U and V represent the distinct sets of nodes within the network, and E denotes the set of relationships or edges connecting these nodes.

The element $\:e=(i,j)$ means that the node $\:i\in\:U$ and the node $\:j\in\:V$ are connected. And the $\:p\text{*}q$ adjacency matrix $\:S$ of the network can be constructed. If $\:(i,j)\in\:E$ exists, then $\:{S}_{ij}=1$, otherwise $\:{S}_{ij}=0$. The key concepts in SNA include:

(1) Unipartite network and bipartite network: The unipartite network focuses on relationships within a single group of actors. The bipartite network involves two distinct sets of actors and examines the relationships between them.

(2) Central index: Degree centrality^17^ is a key measure of the importance of a node in a network based on the number of direct connections it has. The degree centrality *C*_*i*_ of node *i* is the sum of its direct connections to all other nodes. The equation can be expressed as:

2 $${C_i} = \sum\limits_{i = 1}^m {{a_{ij}}} {\text{ }}\left( {i \ne j} \right)$$

where *a*_*ij*_ represents the element in the adjacency matrix *A*, with a value of 1 if node *i* is directly connected to node *j*, and 0 otherwise.

A higher degree of centrality indicates that a node has more connections and plays a more significant role in the network.

### Bow-tie model

The bow-tie model^18^ is a safety assessment method. It establishes a causal graph model with basic events (hazard sources) as the core element and triggers events, barriers, and consequences of basic events as its primary components.

**Fig. 1:**
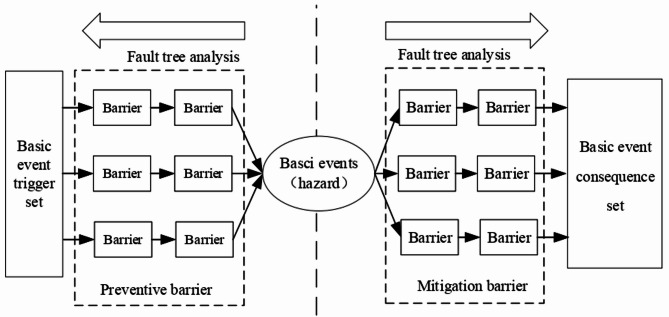
Analysis diagram of bow-tie model.

The structure of the bow-tie model is typically divided into three parts: the fault tree on the left, the event tree on the right, and the essential event in the center. The fault tree represents the initiating events for basic events, while the event tree illustrates the resulting consequences of these basic events. To prevent basic incidents, preventive safety measures (i.e., preventive barriers) are set on the side of the fault tree. Hence, preventive measures are implemented before basic incidents occur. Simultaneously, mitigation safety measures (i.e., mitigation barriers) are positioned on the event tree’s side to implement countermeasures following basic incidents. The schematic diagram of the bow-tie model analysis is presented in Fig. 1.

## Analysis of main causes of coal mine safety risks

### Cause analysis of coal mine safety risks

(1) Access to the cause of the accident.

A total of 72 safety accidents that occurred in a coal mining mine since it began operations are selected for analysis. Then, we prepared an accident questionnaire, including time, place, type of accident, number of casualties, brief history and causes, and accident handling results. Finally, the relevant management personnel are organized to complete the questionnaire.

(2) Handling of accident cause factors.

The data collected from the coal mine safety accident survey are analyzed. The key fields of the questionnaire are extracted, and 85 causal factors are summarized through statistical analysis of the coal mine safety accident data. These causal factors are then numbered, as shown in Table 1.

**Table 1:** Coal mine safety accident factor classification and coding.

Node number	Causation factors	Node number	Causation factors
1	Low skill operation training rate	44	Not constructed as designed
2	Mining mechanization level	45	Integrity rate of mining equipment
3	High incidence of “three violations”	46	Average years of education for technical staff
4	High explosion rate of electromechanical equipment	47	Low rate of hidden danger rectification
5	Inadequate implementation of safety management system	48	Low air volume qualification rate
6	Inadequate safety inspection and hidden danger rectification	49	Air volume supply and demand ratio
7	The imperfection of technical management	50	Carrying fireworks illegally
8	Non-implementation of regulatory orders	51	Obvious fire source
9	Low safety protection facilities	52	Complex geological conditions
10	Average education of years for workers	53	Low intact rate of gas monitoring and monitoring facilities
11	Not operating in accordance with safety standards	54	Other harmful gas control qualification rate
12	Failure to strictly implement gas outburst prevention measures	55	Integrity rate of communication facilities
13	Unqualification electromechanical equipment failure rate	56	Absolute gas emission
14	Poor stability of coal roof and floor	57	Failed to implement the mine inspection system
15	Illegal blasting coal	58	Imperfect monitoring system
16	Failure to strictly implement the gas inspection system	59	Poor safety monitoring system
17	Inadequate implementation of safety education and training	60	Inadequate security measures
18	No corresponding technical measures were taken	61	Low lighting qualification rate
19	No implementation of “three inspections of coal mining by blasting.”	62	Not strict implementation of wind measurement system
20	Low staff employment rate	63	Failed to implement the mine-level leadership system
21	No self-rescuer	64	The imperfection of safety management organization
22	No working conditions	65	Insufficient security staff
23	Improper emergency plan	66	Improper installation location of other equipment
24	Low integrity rate of ventilation facilities	67	Coal dust explosive
25	Proportion of technical personnel	68	Integrity rate of gas drainage facilities
26	Insufficient staffing of special operations	69	No safety production system was established
27	Average wind speed	70	Ground stress
28	Insufficient investment in security technology	71	Low certification rate of operators
29	Poor ventilation of the working face	72	Safety culture
30	Average years of education for managers	73	No “three special"power supply
31	Insufficient attention to safety work	74	Coal seam depth
32	Incomplete implementation of safety measures	75	Weak awareness of mining according to the law
33	Improper construction location	76	Illegal use of explosives and chaotic management
34	Improper technical measures	77	Spontaneous combustion tendency of coal seams
35	Gas concentration	78	Chasm number in the working face
36	Average years of service of technical staff	79	Temperature control qualification rate
37	Residual gas content after drainage	80	Coal seam dip
38	Average years of service of managers	81	Obsolete equipment Installed
39	Seam thickness	82	Gas pressure
40	Inadequate resource supervision	83	No carrying gas detection equipment
41	Serious air leakage	84	Dust control qualification rate
42	Large average fault drops	85	Insufficient understanding of special gas prevention mechanism
43	Raw coal gas content		

Note: Here, we briefly explain some of the professional terms in the coal mine field in the Table 1:.

(1) “Three violations” refers to three types of unsafe behaviors in mining operations: Command violations, Operational violations, and Disciplinary violations.

(2) “Three special power supply” is a safety requirement in coal mining that includes: Special transformer, Special line, and Special person.

(3) “Three Inspections of Coal Mining by Blasting” involves three mandatory inspections: Pre-shift inspection, Inspection during operation, and Post-operation inspection.

### Establishment of coal mine safety risk cause model

Converting bipartite network data into two unipartite network data to investigate the relationship between the elements in each set type is a standard method for analyzing bipartite network data. There are two conversion modes: row mode and column mode. For an accident-cause bipartite network matrix $\:S$, where the number of rows (representing accidents) is *p*, and the number of columns (representing causal factors) is *q*, two 1-modular matrices including accident-accident $\:P$ and cause-cause $\:Q$ can be constructed by using the corresponding product method.

3 $$\:{P}_{ij}=\sum\:_{k=1}^{q}{S}_{ik}{S}_{jk}\:\left(0<i,j\le\:p\right)$$

where

$$\:{S}_{ik}=\left\{\begin{array}{c}0,\:causative\:factor\:k\:not\:in\:accident\:i\\\:1,\:causative\:factor\:k\:\:in\:accident\:i\end{array}\right.$$

$$\:{P}_{ij}=\left\{\begin{array}{c}0,accident\:i\:and\:j\:does\:not\:have\:the\:same\:causative\:factor\\\:m,\left(0<m\le\:p\right),m\:identical\:causes\:exist\:in\:accident\:i\:and\:j\end{array}\right.$$

4 $$\:{Q}_{ij}=\sum\:_{k=1}^{p}{S}_{ki}{S}_{kj}\:\left(0<i,j\le\:q\right)$$

where

$$\:{S}_{kj}=\left\{\begin{array}{c}0,\:causative\:factor\:i\:not\:in\:accident\:k\\\:1,\:causative\:factor\:i\:\:in\:accident\:k\end{array}\right.$$

$$\:{P}_{ij}=\left\{\begin{array}{c}0,causative\:factor\:i\:and\:j\:not\:occur\:together\:in\:any\:accident\\\:n,\left(0<n\le\:p\right),causative\:factor\:i\:and\:j\:appear\:together\:in\:n\:accidents\end{array}\right.$$

By the above method, the accident-cause adjacency matrix can be converted into two 1-modular multi-value matrices — accident-accident $\:P$ and cause-cause $\:Q$, which reflect the strength of the relationship.

The concrete construction method of social network is as follows:

(1) Construction of a bipartite network network matrix.

The accident cause descriptions in the coal mine safety accident report are compared with the accident cause factor classification in Table 1 to construct an accident-factor bipartite network matrix, as shown in Table 2. Each row represents an accident. Each column corresponds to the accident-causing factors in Table 1. If an accident is associated with a causal factor, the corresponding position in the matrix is marked as 1; otherwise, it is recorded as 0.

(2) Construct unipartite network matrix.

To analyze the internal relationships among causal factors in different coal mine safety accidents, a unipartite network network of coal mine safety accident causal factors is constructed to study the co-occurrence relationship of these factors. This paper uses the UCINET software, a widely recognized software package for social network analysis, which has the capacity to create network matrices and inspect the strength of the relationships between the components of the network. Using the column conversion feature in UCINET, the data in Table 2 were converted and the corresponding product method is used to generate the accident cause-accident cause relationship matrix, as shown in Table 3. The number on the diagonal of the 1-modular multi-value matrix represents the occurrence of each causal factor. The off-diagonal entries represent the number of times that causal factors of the corresponding row and column of the element appear together in a coal mine safety accident survey questionnaire. The more significant the co-occurrence of the two factors, the stronger the relationship between them.

**Table 2:** Accident - factor matrix (partial).

factor accident	1	2	3	4	5	6	7	8	9	10
X1	1	1	1	1	1	1	0	0	0	0
X2	0	0	0	0	1	0	1	1	1	0
X3	0	0	0	0	0	1	1	0	0	1
X4	0	0	1	0	0	1	0	1	0	0
X5	0	0	1	0	1	0	1	0	0	0
X6	0	0	1	0	1	0	1	0	0	1
X7	1	0	0	0	0	1	0	1	0	0
X8	0	0	1	0	1	1	0	1	0	0
X9	0	0	1	0	1	1	1	0	0	1
X10	0	0	0	0	1	1	0	0	0	0

**Table 3:** Coal mine safety incident cause factor coexistence matrix (partial).

factor	1	2	3	4	5	6	7	8	9	10
1	2	1	1	1	1	2	0	1	0	0
2	1	9	6	2	6	6	2	4	1	2
3	1	6	33	3	20	13	5	12	1	9
4	1	2	3	4	1	1	0	0	1	0
5	1	6	20	1	39	18	15	13	1	9
6	2	6	13	1	18	28	8	11	0	8
7	0	2	5	0	15	8	20	6	1	4
8	1	4	12	0	13	11	6	20	1	2
9	0	1	1	1	1	0	1	1	2	0
10	0	2	9	0	9	8	4	2	0	14

5 $$\:{w}_{ij}=\frac{n(i,j)}{72}$$

(3) Edge weights setting.

The connection strength, referred to as the edge weight, is the proportion of accidents in which the two causal factors co-occur relative to the total number of accidents. Assuming that causal factors $\:i$ and $\:j$ occur together in $\:n$ coal mine safety accidents, where $\:n(i,j)$ represents the number of accidents involving both causal factors $\:i$ and $\:j$. The formula for calculating the edge weight $\:{w}_{ij}$ between two nodes is as follows:.

**Fig. 2:**
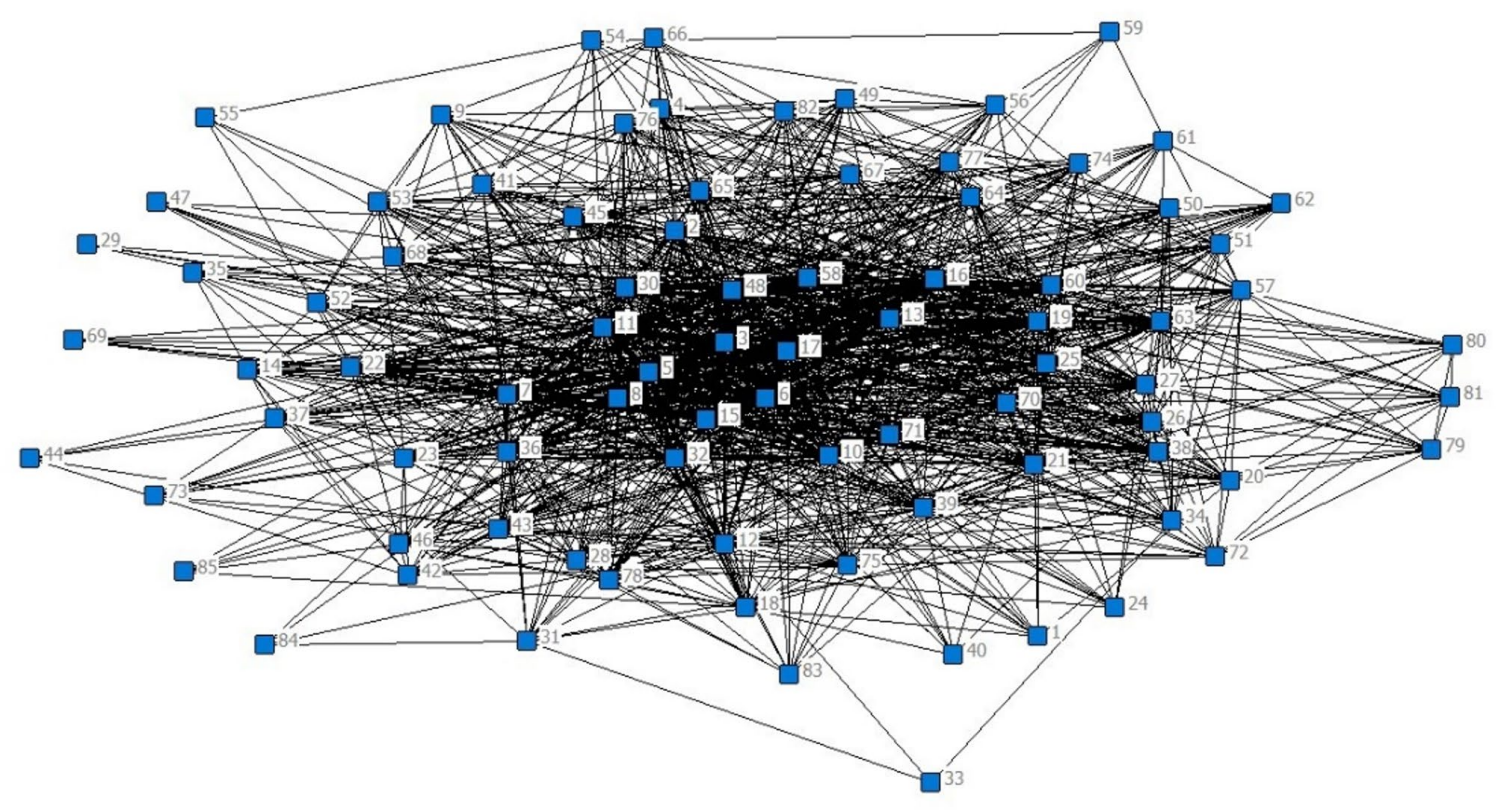
Network model of coal mine safety accident cause factors.

The SNA structure diagram of coal mine safety causes is shown in Fig. 2. The nodes in the figure represent the causal factors, and the edges between them indicate the co-occurrence or the strength of their association in safety accidents. The size of each node corresponds to the frequency of its occurrence as a contributing factor in the accidents, while the edge weights between nodes represent the frequency of simultaneous occurrence of these factors across the dataset. The strength of the relationships between factors is determined by the edge weights, calculated as the proportion of accidents where two factors co-occur, as shown in Eq. 5. The thicker the edge between two nodes, the stronger the correlation between the corresponding causal factors.

To analyze relationships among the causal factors of coal mine safety accidents, a unipartite network of these factors was established. It converted the original accident-cause matrix into a cause-cause relationship matrix, capturing the strength of co-occurrence among the causal factors. The resulting network model is illustrated in Fig. 2, where nodes represent the identified 85 causal factors, and edges indicate the existence of co-occurrences between pairs of causal factors. The structural relationships illustrated in the network serve as a qualitative foundation, while the relative importance and interactions among causal factors are evaluated through frequency counts, co-occurrence intensities, and network centrality metrics.

### Social network analysis results

(1) Node frequency analysis.

The frequency analysis was conducted on nodes representing causal factors within the network illustrated in Fig. 2. The top 10 nodes, ranked by their frequency of occurrence across the 72 analyzed coal mine safety accidents, are presented in Table 4. The most frequent causal factor identified is “Inadequate implementation of safety management systems” (Node 5), appearing in 39 accidents. Similarly prevalent is the factor “High incidence of three violations” (Node 3), associated with 33 incidents. These findings underscore the critical influence of human and management-related factors, highlighting the necessity of addressing these dimensions in coal mine safety risk management strategies.

**Table 4:** Top 10 of coal mine safety accident factor analysis.

Node number	Causation factors	Frequency
5	Inadequate implementation of safety management system	39
3	High incidence of “three violations”	33
11	Not operating in accordance with safety standards	29
6	Inadequate safety inspection and hidden danger rectification	28
17	Inadequate implementation of safety education and training	24
7	The imperfection of technical management	20
8	Non-implementation of regulatory orders	20
15	Illegal blasting coal	17
13	Unqualification electromechanical equipment failure rate	15
10	Poor safety awareness of workers	14
16	Failure to strictly implement the gas inspection system	14

(2) Edge weight analysis.

In the analysis of the edges within the coal mine safety accident cause factor network, the weights of the 1,223 connections are quantified. The top ten edge weights, presented in Table 5, are selected by sorting the edge weights in descending order. In the coal mine safety accident cause network shown in Fig. 2, the connection between nodes 3 and 5 is the thickest. In the coal mine safety accident cause network depicted in Fig. 2, the connection between nodes 3 and 5 is the thickest. The edge weight reaches 0.278, indicating that the accident cause factors represented by these two nodes frequently co-occur in coal mine safety accidents, contributing to the occurrence of accidents in conjunction with other factors.

**Table 5:** Top 10 of the right of coal mine safety incident causation factors network.

Representation of edges	Causation factors 1	Causation factors 2	Edge weight
3–5	High incidence of “three violations”	Inadequate implementation of safety management system	0.278
5–6	Inadequate implementation of safety management system	Inadequate safety inspection and hidden danger rectification	0.250
5–17	Inadequate implementation of safety management system	Inadequate implementation of safety education and training	0.236
5–11	Inadequate implementation of safety management system	Not operating in accordance with safety standards	0.222
5–7	Inadequate implementation of safety management system	The imperfection of technical management	0.208
3–11	High incidence of “three violations”	Not operating in accordance with safety standards	0.194
3–17	High incidence of “three violations”	Inadequate implementation of safety education and training	0.194
3–6	High incidence of “three violations”	Inadequate safety inspection and hidden danger rectification	0.181
5–8	Inadequate implementation of safety management system	Non-implementation of regulatory orders	0.181
3–8	High incidence of “three violations”	Non-implementation of regulatory orders	0.1667

By analyzing the connections between factors in coal mine safety accidents, we can sever edges with higher weights in the network to prevent the two connected node factors from working together. For example, reducing the connection between Nodes 3 and 5, which represent high violation rates and poor safety management, may help prevent many safety incidents. Table 5 emphasizes that focusing on edges with higher weights could be an effective strategy for reducing the overall frequency of coal mine accidents. This shows that focusing on edges with higher weights could be an effective strategy for reducing the overall frequency of coal mine accidents.

(3) Node centrality analysis.

In social network analysis, degree centrality is a critical measure to identify the most influential nodes within a network. We analyzed the degree centrality of the coal mine safety accident cause factors using UCINET software, and the results are presented in Table 6. The degree centrality of Nodes 11 (“Not operating in accordance with safety standards”) and 6 (“Inadequate safety inspection and hidden danger rectification”) is the highest, both at 68. This indicates that these factors are highly influential within the safety accident network and frequently co-occur with other accident causes, contributing significantly to the overall risk profile of coal mining operations.

**Table 6:** Central indicator of some nodes of coal mine safety incident causation factors network.

Node number	Causation factors	Central indicator
11	Not operating in accordance with safety standards	68
6	Inadequate safety inspection and hidden danger rectification	68
5	Inadequate implementation of safety management system	64
17	Inadequate implementation of safety education and training	64
13	Unqualification electromechanical equipment failure rate	62
3	High incidence of “three violations”	61
16	Failure to strictly implement the gas inspection system	60
8	Non-implementation of regulatory orders	56
48	Low air volume qualification rate	54
7	The imperfection of technical management	54

The nodes with the highest degree centrality are predominantly related to human factors such as safety violations, lack of safety management, and insufficient training. This aligns with our earlier findings that human error and systemic management failures are key contributors to coal mine accidents. The high centrality of these nodes suggests that focusing on improving safety management practices, training programs, and regulatory compliance could have a significant impact on overall safety.

## Management and control of coal mine safety risk causes based on Bow-tie model

### Classification of causes of coal mine safety risks

The effective management of the causes of coal mine safety risks is a crucial measure for ensuring the intrinsic safety of coal mine operations. Through the analysis of coal mine safety risk using social networks, the main causes can be counted regarding the number of causative factors, edge weights, and degree of centrality. The statistical results are shown in Table 7.

**Table 7:** Frequency statistics of coal mine safety incident causation factors.

Causation factors	Frequency	Causation factors	Frequency
Inadequate implementation of safety management system	8	The imperfection of technical management	3
High incidence of “three violations”	7	Failure to strictly implement the gas inspection system	2
Inadequate safety inspection and hidden danger rectification	4	Unqualification electromechanical equipment failure rate	2
Not operating in accordance with safety standards	4	Low air volume qualification rate	1
Inadequate implementation of safety education and training	4	Poor safety awareness of workers	1
Non-implementation of regulatory orders	4	Illegal blasting coal	1

Table 7 shows 12 main causes of coal mine safety accidents. The frequency of inadequate implementation of safety management systems is the highest, reaching 8 times. Other causes, such as low air volume qualification rates, poor safety awareness among workers, and illegal coal blasting, each occur once. The main causal factors are classified and divided according to the four “human”, “machine”, “environment”, and “management” levels in the traditional coal mine safety evaluation index system^19^.

Human factors include inadequate implementation of the safety management system, a high incidence of “three violations”, failure to operate according to safety standards, non-implementation with regulatory orders, failure to strictly implement the gas inspection system, poor safety awareness among workers, and illegal coal blasting. Equipment factors include the failure of electromechanical equipment to meet qualification standards. Environmental factors include a low air volume qualification rate. Management factors include inadequate safety inspection and hidden danger rectification, inadequate safety education and training, and imperfection of technical management.

### Construction of Bow-tie model of coal mine safety risk

Based on the above analysis, human factors are the main cause of coal mine safety accidents. The combination of management, environmental, and equipment factors plays a secondary role. The interaction between main and secondary factors leads to coal mine safety accidents. Therefore, human, equipment, environmental, and management factors are defined as the top (essential) events in the bow-tie mode. In combination with the cause classification results, the four types of causes, results, and related control measures are determined to prevent the occurrence of top-level events.

**Fig. 3:**
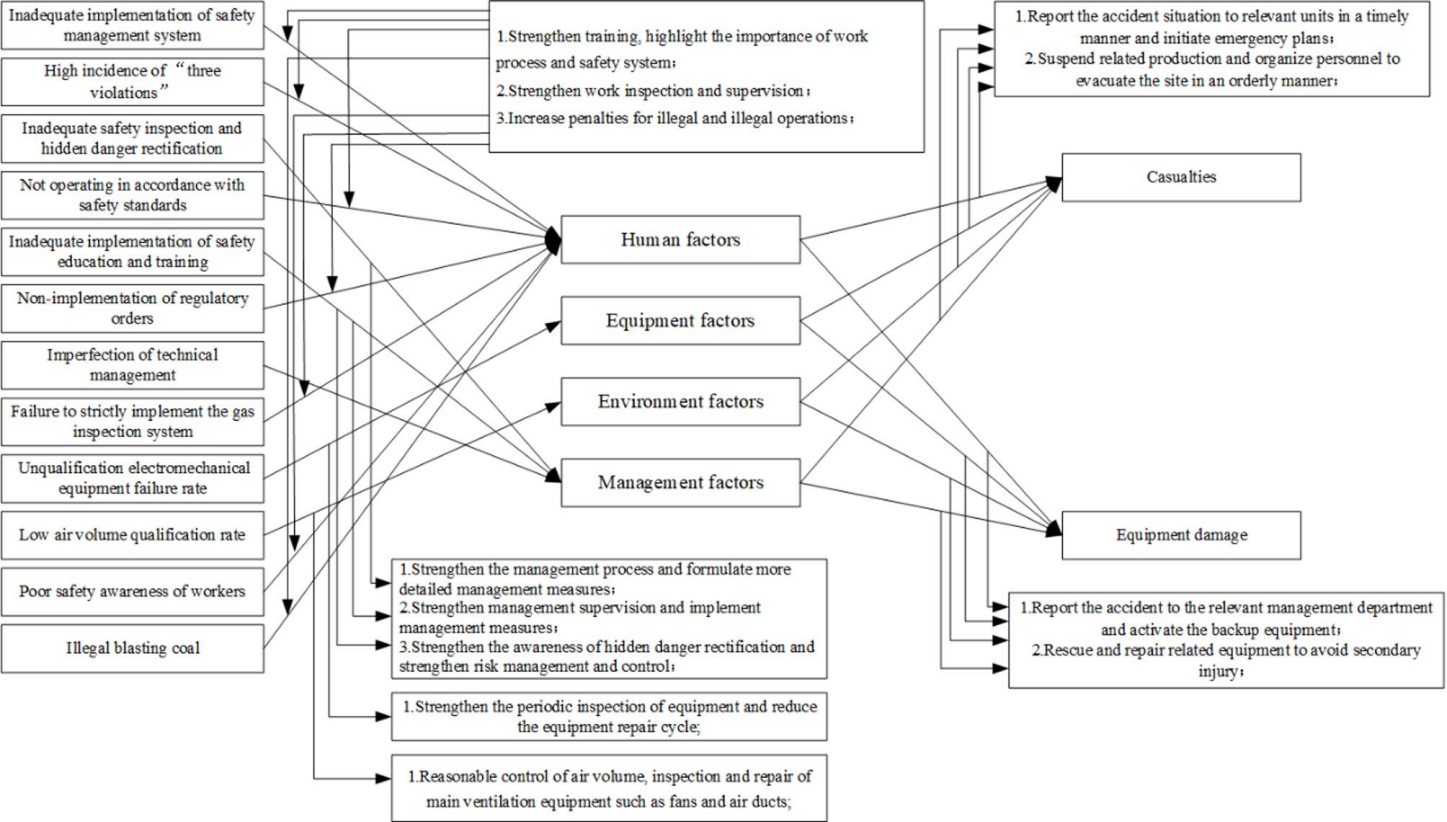
Bow-tie model of coal mine safety risk.

According to the theory of hazard sources 19, coal mine safety accidents generally proceed through the following stages. The first stage is the failure of risk control measures for the inherent hazard sources in coal mines, which results in hidden safety hazards. The second stage is the failure of coal mine safety hazards to be rectified in a timely manner, which leads to the transformation of hidden dangers into actual accidents. Therefore, after fully identifying the inherent hazards in coal mines, the primary preventive measure is the timely elimination of various hidden dangers. Due to the particularity of the disasters caused by the complex coal mine production environment, the safety measures during coal mine accidents primarily focus on timely rescue operations, the scientific formulation of rescue plans, and the aftermath when control measures fail. In summary, the construction of the bow-tie model for coal mine safety risk is completed, as illustrated in Fig. 3.

### Association rule mining of gas safety risk causation factors in coal mines

In coal mine gas safety risk analysis, individual causative factors rarely act in isolation. Instead, accidents are often the result of complex interactions among multiple contributing factors. Identifying these latent interdependencies is essential for comprehensive risk prevention. To this end, this study employs the Apriori algorithm to conduct association rule mining on the gas safety accident dataset, aiming to uncover frequent co-occurrences and strong associations among causative factors.

The Apriori algorithm is a classical data mining technique that identifies association rules based on user-specified minimum thresholds of support and confidence. In this study, the accident-factor matrix was converted into a transaction format compatible with the algorithm, and the minimum support and confidence were set to 0.05 and 0.80, respectively. The core evaluation metrics are defined as follows:

6 $$\begin{gathered} Support(A \to B) = P(A \cap B) = \frac{{{\text{count}}(A \cap B)}}{{{\text{count}}(T)}} \hfill \\ Confidence(A \to B) = P(B|A) = \frac{{{\text{count}}(A \cap B)}}{{{\text{count}}(A)}} \hfill \\ \end{gathered}$$

where ${\text{count}}(A \cap B)$ is the number of transactions containing both itemsets *A* and *B*, and ${\text{count}}(T)$ is the total number of transactions.

The association rule mining yielded a total of 73 high-confidence rules, a subset of which are shown in Table 8. These rules capture significant patterns of co-occurrence among causative factors, particularly those related to gas safety risks.

**Table 8:** Mining results of coal mine gas safety accident causation factors association rules.

No.	Association Rule (Textual)	Support	Confidence	Lift
1	{Poor stability of coal roof and floor} ⇒ {Inadequate implementation of safety management system}	0.0556	1	1.8462
2	{Air volume supply and demand ratio} ⇒ {High incidence of “three violations”}	0.0556	0.8	1.7455
3	{Average years of service of managers} ⇒ {Inadequate safety inspection and hidden danger rectification}	0.0556	1	2.5714
4	{Integrity rate of gas drainage facilities} ⇒ {Low air volume qualification rate}	0.0556	0.8	3.84
5	{Integrity rate of gas drainage facilities} ⇒ {High incidence of “three violations”}	0.0556	0.8	1.7455
6	{Failed to implement the mine inspection system} ⇒ {Failure to strictly implement the gas inspection system}	0.0556	1	5.1429
7	{Chasm number in the working face} ⇒ {Inadequate implementation of safety education and training}	0.0556	0.8	2.4
8	{Improper emergency plan} ⇒ {Inadequate safety inspection and hidden danger rectification}	0.0694	0.8333	2.1429
9	{Improper emergency plan} ⇒ {Inadequate implementation of safety management system}	0.0694	0.8333	1.5385
10	{Ground stress} ⇒ {Failure to strictly implement the gas inspection system}	0.0694	0.8333	4.2857
11	{Proportion of technical personnel} ⇒ {Inadequate safety inspection and hidden danger rectification}	0.0833	0.8571	2.2041
12	{Inadequate safety inspection and hidden danger rectification, Improper emergency plan} ⇒ {High incidence of “three violations”}	0.0556	0.8	1.7455
13	{High incidence of “three violations”, Improper emergency plan} ⇒ {Inadequate safety inspection and hidden danger rectification}	0.0556	1	2.5714
14	{Inadequate safety inspection and hidden danger rectification, Improper emergency plan} ⇒ {Inadequate implementation of safety management system}	0.0694	1	1.8462
15	{Inadequate implementation of safety management system, Improper emergency plan} ⇒ {Inadequate safety inspection and hidden danger rectification}	0.0694	1	2.5714
16	{High incidence of “three violations”, Improper emergency plan} ⇒ {Inadequate implementation of safety management system}	0.0556	1	1.8462
17	{Inadequate implementation of safety management system, Improper emergency plan} ⇒ {High incidence of “three violations”}	0.0556	0.8	1.7455
18	{Inadequate implementation of safety education and training, Average years of service of technical staff} ⇒ {High incidence of “three violations”}	0.0556	0.8	1.7455
19	{Inadequate safety inspection and hidden danger rectification, Average years of service of technical staff} ⇒ {Inadequate implementation of safety management system}	0.0556	0.8	1.4769
20	{High incidence of “three violations”, Average years of service of technical staff} ⇒ {Inadequate implementation of safety management system}	0.0694	0.8333	1.5385
21	{Inadequate implementation of safety management system, Average years of service of managers} ⇒ {High incidence of “three violations”}	0.0694	0.8333	1.8182
22	{Mining mechanization level, Non-implementation of regulatory orders} ⇒ {Inadequate implementation of safety management system}	0.0556	1	1.8462
23	{Mining mechanization level, High incidence of “three violations”} ⇒ {Inadequate implementation of safety management system}	0.0694	0.8333	1.5385
24	{Mining mechanization level, Inadequate implementation of safety management system} ⇒ {High incidence of “three violations”}	0.0694	0.8333	1.8182
25	{Failure to strictly implement the gas inspection system, Proportion of technical personnel} ⇒ {Inadequate safety inspection and hidden danger rectification}	0.0556	1	2.5714
26	{Inadequate implementation of safety education and training, Proportion of technical personnel} ⇒ {Inadequate safety inspection and hidden danger rectification}	0.0694	1	2.5714
27	{Inadequate safety inspection and hidden danger rectification, Proportion of technical personnel} ⇒ {Inadequate implementation of safety education and training}	0.0694	0.8333	2.5
28	{Non-implementation of regulatory orders, No implementation of “three inspections of coal mining by blasting”} ⇒ {Illegal blasting coal}	0.0556	0.8	3.3882
29	{Unqualification electromechanical equipment failure rate, Average years of education for managers} ⇒ {Inadequate implementation of safety education and training}	0.0556	0.8	2.4
30	{Unqualification electromechanical equipment failure rate, Average years of education for managers} ⇒ {Inadequate implementation of safety management system}	0.0556	0.8	1.4769
31	{Not operating in accordance with safety standards, Average years of education for managers} ⇒ {Inadequate implementation of safety education and training}	0.0556	0.8	2.4
32	{Inadequate safety inspection and hidden danger rectification, Average years of education for managers} ⇒ {Inadequate implementation of safety education and training}	0.0694	1	3
33	{Inadequate implementation of safety education and training, Average years of education for managers} ⇒ {Inadequate implementation of safety management system}	0.0833	0.8571	1.5824
34	{Inadequate safety inspection and hidden danger rectification, Average years of education for managers} ⇒ {Inadequate implementation of safety management system}	0.0556	0.8	1.4769
35	{The imperfection of technical management, Complex geological conditions} ⇒ {Inadequate implementation of safety management system}	0.0694	1	1.8462
36	{Inadequate implementation of safety education and training, Complex geological conditions} ⇒ {Inadequate safety inspection and hidden danger rectification}	0.0556	0.8	2.0571
37	{The imperfection of technical management, Low air volume qualification rate} ⇒ {Inadequate implementation of safety management system}	0.0556	0.8	1.4769
38	{High incidence of “three violations”, Low air volume qualification rate} ⇒ {Inadequate implementation of safety education and training}	0.0556	0.8	2.4
39	{Inadequate implementation of safety education and training, Low air volume qualification rate} ⇒ {Inadequate implementation of safety management system}	0.0833	0.8571	1.5824
40	{Poor safety awareness of workers, Illegal blasting coal} ⇒ {Inadequate implementation of safety management system}	0.0556	1	1.8462
41	{Poor safety awareness of workers, Inadequate implementation of safety education and training} ⇒ {Inadequate implementation of safety management system}	0.0556	1	1.8462
42	{Unqualification electromechanical equipment failure rate, Inadequate implementation of safety education and training} ⇒ {Inadequate implementation of safety management system}	0.0556	1	1.8462
43	{Inadequate safety inspection and hidden danger rectification, Inadequate implementation of safety education and training} ⇒ {Inadequate implementation of safety management system}	0.0694	0.8333	1.5385
44	{Inadequate implementation of safety management system, Inadequate implementation of safety education and training} ⇒ {High incidence of “three violations”}	0.0694	0.8333	1.8182
45	{Inadequate implementation of safety management system, Inadequate implementation of safety education and training} ⇒ {Inadequate safety inspection and hidden danger rectification}	0.0694	0.8333	2.1429
46	{High incidence of “three violations”, Inadequate implementation of safety education and training} ⇒ {Inadequate safety inspection and hidden danger rectification}	0.0694	1	2.5714
47	{High incidence of “three violations”, Inadequate implementation of safety education and training} ⇒ {Inadequate implementation of safety management system}	0.0833	1	1.8462
48	{High incidence of “three violations”, Inadequate safety inspection and hidden danger rectification} ⇒ {Inadequate implementation of safety management system}	0.0833	1	1.8462
49	{Inadequate implementation of safety management system, Inadequate safety inspection and hidden danger rectification} ⇒ {High incidence of “three violations”}	0.0833	1	2.1818
50	{Inadequate implementation of safety management system, Inadequate safety inspection and hidden danger rectification} ⇒ {Inadequate implementation of safety education and training}	0.0694	0.8333	2.5
51	{High incidence of “three violations”, Inadequate implementation of safety management system} ⇒ {Inadequate safety inspection and hidden danger rectification}	0.0833	1	2.5714
52	{High incidence of “three violations”, Inadequate implementation of safety management system} ⇒ {Inadequate implementation of safety education and training}	0.0694	0.8333	2.5
53	{High incidence of “three violations”, Inadequate safety inspection and hidden danger rectification} ⇒ {Inadequate implementation of safety education and training}	0.0694	0.8333	2.5
54	{High incidence of “three violations”, Inadequate safety inspection and hidden danger rectification, Inadequate implementation of safety education and training} ⇒ {Inadequate implementation of safety management system}	0.0694	1	1.8462
55	{Inadequate implementation of safety management system, Inadequate safety inspection and hidden danger rectification, Inadequate implementation of safety education and training} ⇒ {High incidence of “three violations”}	0.0694	1	2.5714
56	{Inadequate implementation of safety management system, Inadequate safety inspection and hidden danger rectification, Inadequate implementation of safety education and training} ⇒ {High incidence of “three violations”}	0.0694	1	2.1818
57	{High incidence of “three violations”, Inadequate safety inspection and hidden danger rectification, Inadequate implementation of safety education and training} ⇒ {Inadequate implementation of safety education and training}	0.0694	0.8333	2.5
58	{High incidence of “three violations”, Inadequate safety inspection and hidden danger rectification, Inadequate implementation of safety education and training} ⇒ {Inadequate implementation of safety education and training}	0.0694	0.8333	2.5
59	{High incidence of “three violations”, Inadequate implementation of safety education and training, Inadequate implementation of safety management system} ⇒ {Inadequate safety inspection and hidden danger rectification}	0.0694	1	2.5714
60	{High incidence of “three violations”, Inadequate safety inspection and hidden danger rectification, Inadequate implementation of safety education and training} ⇒ {Inadequate implementation of safety management system}	0.0694	1	1.8462
61	{Inadequate implementation of safety management system, Inadequate safety inspection and hidden danger rectification, Inadequate implementation of safety education and training} ⇒ {High incidence of “three violations”}	0.0694	1	2.1818
62	{Inadequate implementation of safety management system, Inadequate safety inspection and hidden danger rectification, Inadequate implementation of safety education and training} ⇒ {High incidence of “three violations”}	0.0694	1	2.1818
63	{Inadequate implementation of safety management system, Inadequate safety inspection and hidden danger rectification, Inadequate implementation of safety education and training} ⇒ {High incidence of “three violations”}	0.0694	1	2.1818
64	{Inadequate implementation of safety management system, Inadequate safety inspection and hidden danger rectification, Inadequate implementation of safety education and training} ⇒ {High incidence of “three violations”}	0.0694	1	2.1818
65	{Inadequate implementation of safety management system, Inadequate safety inspection and hidden danger rectification, Inadequate implementation of safety education and training} ⇒ {High incidence of “three violations”}	0.0694	1	2.1818
66	{Inadequate implementation of safety management system, Inadequate safety inspection and hidden danger rectification, Inadequate implementation of safety education and training} ⇒ {High incidence of “three violations”}	0.0694	1	2.1818
67	{Inadequate implementation of safety management system, Inadequate safety inspection and hidden danger rectification, Inadequate implementation of safety education and training} ⇒ {High incidence of “three violations”}	0.0694	1	2.1818
68	{Inadequate implementation of safety management system, Inadequate safety inspection and hidden danger rectification, Inadequate implementation of safety education and training} ⇒ {High incidence of “three violations”}	0.0694	1	2.1818
69	{Inadequate implementation of safety management system, Inadequate safety inspection and hidden danger rectification, Inadequate implementation of safety education and training} ⇒ {High incidence of “three violations”}	0.0694	1	2.1818
70	{Inadequate implementation of safety management system, Inadequate safety inspection and hidden danger rectification, Inadequate implementation of safety education and training} ⇒ {High incidence of “three violations”}	0.0694	1	2.1818
71	{Inadequate implementation of safety management system, Inadequate safety inspection and hidden danger rectification, Inadequate implementation of safety education and training} ⇒ {High incidence of “three violations”}	0.0694	1	2.1818
72	{Inadequate implementation of safety management system, Inadequate safety inspection and hidden danger rectification, Inadequate implementation of safety education and training} ⇒ {High incidence of “three violations”}	0.0694	1	2.1818
73	{Inadequate implementation of safety management system, Inadequate safety inspection and hidden danger rectification, Inadequate implementation of safety education and training} ⇒ {High incidence of “three violations”}	0.0694	1	2.1818

Table 8 shows the mining results of association rules for the causes of coal mine gas accidents. It can be found that several causative factors, including inadequate safety education and training, insufficient safety inspections and hidden danger rectification, high incidence of “three violations”, poor implementation of safety management systems, and failure to enforce gas inspection protocols, appear frequently and prominently as consequents across multiple rules. The relatively high number of associations pointing to these nodes indicates that they are critical contributing factors in gas-related accidents and play a central role in the formation of accident-prone conditions. Additionally, Rules 4, 28, and 63 show lift values exceeding 3 and involve factors such as ventilation system integrity, adherence to standardized operating procedures, and the installation rate of monitoring and control facilities, further underscoring their importance in coal mine gas safety.

Moreover, the rules in which the consequent is the failure to enforce gas inspection procedures exhibit particularly high lift values, with Rules 6 and 69 both exceeding a lift of 5. These rules involve precursors such as the failure to conduct pre-entry gas inspections and deficiencies in technical management, suggesting that insufficient oversight in these areas is often accompanied by inadequate enforcement of gas inspection protocols. Furthermore, rules which demonstrate insufficient safety education and training, such as Rules 32, 58, and 68, have also been found to have elevated lift values, with all values exceeding 3. These rules are associated with managerial negligence in educational oversight, indicating that systemic managerial lapses often lead to ineffective or superficial training practices.

These findings reinforce the conclusion that coal mine gas safety accidents are rarely the result of isolated failures. Instead, they reflect systemic deficiencies in management, supervision, and operational compliance.

## Discussion

On one hand, the practical analysis of coal mine safety risk causative factors can reveal the hazard sources of coal mine safety accidents. On the other hand, safety management measures targeting risk sources can be developed, which ensures the safety of coal mine production and significantly enhancing the level of coal mine safety management. This article analyzes coal mine risk causative factors and their management model, and will discuss the following sections:

Firstly, a comprehensive list of 85 causative factors was extracted from a total of 72 accidents in coal mine enterprises Based on the SNA, a coal mine safety risk causes network is constructed. The analysis reveals that management factors are the main causative factors of coal mine safety accidents. Therefore, managing the risks associated with management factors will be crucial to reducing coal mine safety accidents.

Secondly, we design a questionnaire with preprocessed keywords to obtain these coal mine causative factors. However, different experts provide varying descriptions in the original questionnaire. During processing, the meaning of some expressions may change. To avoid misinterpretation, the keywords in the questionnaire should be standardized in future research. On the other hand, coal mine accidents are affected by various factors, such as area, time, mining depth, and so on. Some factors are not included in the questionnaire. In other words, the analysis of accident factors is not comprehensive due to cognitive limitations. A more extensive summary and analysis of influencing factors will also be a part of future research.

Finally, with the development of smart mines, integrating safety management theory with smart mines will facilitate the construction of coal mines. During the construction process, safety management displayed in information will be a challenge. Based on the current state of coal mine informatization, it is feasible to apply algorithms in line with coal mine characteristics for coal mine safety management processing data. Subsequently, data analysis results can be interpreted and analyzed using the coal mine safety management theory, which can enhance safety management practices and decrease the probability of accidents.

## Conclusions

(1) Based on the coal mine safety risk accident data, 85 coal mine safety risk causes are extracted. The indicators are analyzed using the three levels of frequency, edge weight, and centrality indicators based on the SNA method. The main coal mine safety risks are clarified, and a set of leading causes for these risks is established.

(2) Association rules among gas safety risk factors were mined using the Apriori algorithm. The results demonstrate frequent co-occurrence and mutual reinforcement among factors such as high incidence of “three violations”, inadequate safety management, insufficient safety inspection, and deficient safety education—validating that multi-factor coupling is a critical mechanism underlying accident occurrence.

(3) The causative factors were classified into human, equipment, environmental, and management categories. A bow-tie model was constructed to visualize the risk transmission pathways and formulate targeted control measures at each stage. The integration of association rule mining enhances this model by providing quantitative evidence of high-risk cause combinations, enabling more precise prevention strategies.

(4) Coal mine safety accidents stem from not just individual risk factors but from their complex interactions. Strengthening safety management systems, enforcing standard operating procedures, improving safety training, and addressing multi-factor synergies are essential for comprehensive risk mitigation.

## Data Availability

All data generated or analysed during this study are included in this published article.

## References

[1] Cao, Q. R. & Xu, Z. Q. The behavioral causation chain of coal production accidents and its prevention and control measures. *China Saf. Sci. J.* **20** (09), 127–131 (2010).

[2] Zhang, J. M. Coal mine accident causation theory and safety management measures. *China Coal*. **39** (06), 93–97 (2013).

[3] Zhang, D., NIE, B. S. & WANG, L. K. Investigation a disaster-causing factors of coal mine accidents in China. *J. Saf. Sci. Tech.* **9** (05), 136–140 (2013).

[4] Li, X. G., GE, J. J., HU, T., LU, J. & PAN, K. K. Bayesian network modeling for causation analysis of coal mine roof accident. *China Saf. Sci. J.* **24** (07), 10–14 (2014).

[5] Qiao, W. G. *Analysis of Mine Accident Risk Factor Coupling and Simulation of Coupling Risk[Master]* (Xuzhou:China University of Mining and Technology, 2014).

[6] Liu, Q. L., Li, X. C. & Wang, L. Analysis and measurement of risk factors coupling in coal mine accidents. *Statics Inform. Forum*. **30** (03), 82–87 (2015).

[7] Zhang, C., Wang, Z. T., YANG, D. Y. & Tian, C. C. Study on mine safety manufacture accidents by factorial analysis. *Coal Technol.* **35** (06), 314–315 (2016).

[8] Tan, Z. L. & Song, Q. Z. Analysis of coal mine safety accidents based on grounded theory. *Saf. Coal Mines*. **48** (09), 238–240 (2017).

[9] Wang, Y. X., Fu, G. & Qian, L. et al. Accident case-driven study on the causal modeling and prevention strategies of coal-mine gas-explosion accidents: A systematic analysis of coal-mine accidents in China. *Resour. Policy* **88**, 104425 (2024).

[10] Fa, Z. et al. From correlation to causality: Path analysis of accident-causing factors in coal mines from the perspective of human, machinery, environment and management. *Resour. Policy* **73**, 102157 (2021).

[11] Fu, G. et al. The development history of accident causation models in the past 100 years: 24Model, a more modern accident causation model. *Process Saf. Environ. Prot.* **134**, 47–82 (2020).

[12] Wang, Y. et al. Modelling and analysis of unsafe acts in coal mine gas explosion accidents based on network theory. *Process Saf. Environ. Prot.* **170**, 28–44 (2023).

[13] Zhang, J. J. et al. Study on causation mechanism of extraordinary serious gas explosion accidents in coal mines. *China Saf. Sci. J.* **27**(01), 48–52 (2017).

[14] Wang, X. F. Study on causing factors of coal mine accident based on support vector machine and fuzzy-Bayesian method. (China University of Mining and Technology, Xuzhou, China, 2019).

[15] He, G., Zhu, Y. N., Zhang, G. S. & Qiao, G. T. Coupling model for formation cause of coal mine employees’ safety risk based on SEM. *Min. Saf. Environ. Prot.* **43**(06), 103–106 (2016).

[16] Li, S. Q., Feng, Y. Q., Hu, S. H., Feng, L. X. Research on relationship among various kinds of unsafe construction behavior based on social network analysis. *China Saf. Sci. J.* **27**(06), 7–12 (2017).

[17] Cao, Z., Wang, T., Lv, L. H. & Xie, Y. S. Research on safety assessment of LPG filling-station based on social network analysis. *China Saf. Sci. J.* **25**(03), 71–77 (2015).

[18] Xu, Q. & Xu, K. Mine safety assessment using gray relational analysis and bow tie model. *PLoS ONE* **13**(3), e0193576 (2018). 29561875 10.1371/journal.pone.0193576 PMC5862416

[19] Li, H. & Fu, G. Reanalysis on meaning of hazards. *China Saf. Sci. J.* **29** (07), 1–5 (2019).

